# Dissecting the role of adult hippocampal neurogenesis towards resilience versus susceptibility to stress-related mood disorders

**DOI:** 10.1038/s41539-022-00133-y

**Published:** 2022-07-16

**Authors:** Katherine L. Jones, Mei Zhou, Dhanisha J. Jhaveri

**Affiliations:** 1 Queensland Brain Institute, University of Queensland, Brisbane, QLD 4072 Australia; 2 Mater Research Institute - University of Queensland, Translational Research Institute, Woolloongabba, QLD 4102 Australia

**Keywords:** Adult neurogenesis, Cellular neuroscience

## Abstract

Adult hippocampal neurogenesis in the developmental process of generating and integrating new neurons in the hippocampus during adulthood and is a unique form of structural plasticity with enormous potential to modulate neural circuit function and behaviour. Dysregulation of this process is strongly linked to stress-related neuropsychiatric conditions such as anxiety and depression, and efforts have focused on unravelling the contribution of adult-born neurons in regulating stress response and recovery. Chronic stress has been shown to impair this process, whereas treatment with clinical antidepressants was found to enhance the production of new neurons in the hippocampus. However, the precise role of adult hippocampal neurogenesis in mediating the behavioural response to chronic stress is not clear and whether these adult-born neurons buffer or increase susceptibility to stress-induced mood-related maladaptation remains one of the controversial issues. In this review, we appraise evidence probing the causal role of adult hippocampal neurogenesis in the regulation of emotional behaviour in rodents. We find that the relationship between adult-born hippocampal neurons and stress-related mood disorders is not linear, and that simple subtraction or addition of these neurons alone is not sufficient to lead to anxiety/depression or have antidepressant-like effects. We propose that future studies examining how stress affects unique properties of adult-born neurons, such as the excitability and the pattern of connectivity during their critical period of maturation will provide a deeper understanding of the mechanisms by which these neurons contribute to functional outcomes in stress-related mood disorders.

## Introduction

The discovery of adult neurogenesis, i.e. the birth of new neurons in the adult brain, transformed our understanding of the extent of structural plasticity in the adult nervous system and heralded a new era in regenerative medicine by highlighting potential brain repair strategies for the treatment of neurodegenerative and neuropsychiatric conditions. The long-held dogma of a hard-wired, immutable brain was initially challenged by Joseph Altman and colleagues in the 1960s and pioneering studies by his group provided the first evidence supporting the generation of new neurons in the adult rodent brain^1,2^. However, these findings were largely dismissed due to the dominant views at the time and failed attempts by others to replicate their findings^3–5^. The field then remained dormant for the next two decades before its resurgence in the 1990s, when advances in immunolabelling and microscopy, as well as cell culture, approaches firmly established the production and integration of new neurons in the rodent brain (reviewed in detail in refs. ^6,7^). The multi-stage process of adult neurogenesis was shown to encompass the proliferation of endogenous populations of neural precursor cells, their differentiation into neurons and their migration and integration into existing neural circuitry^7,8^. This was found to occur primarily in two regions of the adult brain—the subventricular zone of the lateral ventricles and the subgranular zone of the dentate gyrus in the hippocampus in most mammals^9^. Subsequently, several other regions, including the basolateral amygdala^10,11^ and hypothalamus^12,13^, were also shown to harbour this neurogenic capacity, albeit to a limited extent.

The hippocampus is an important brain region involved in the regulation of cognitive processes such as learning and memory as well as mood^14^. Studies in animal models have provided overwhelming support for the role of adult hippocampal neurogenesis in spatial and contextual learning, memory and forgetting (for details, see reviews^15–17^). However, its role in mood regulation is not fully understood. Given that deficits in learning and memory are common to neuropsychiatric disorders, it has been suggested that adult-born neurons could contribute to the pathophysiology of these disorders indirectly via the regulation of cognitive functions^18,19^. Thus, the potential of these adult-born neurons to impact fundamental brain functions spurred immense interest in establishing the occurrence and extent of neurogenesis in the adult human hippocampus. An initial birth-dating study using BrdU (5-Bromo-2′-deoxyuridine) to label dividing cells^20^, and a subsequent 14 C dating study^21^ provided tantalising evidence supporting the generation of new neurons in the adult human hippocampus. Although there are no approaches to directly and reliably measure levels of adult hippocampal neurogenesis in the live human brain, these findings have been corroborated by immunohistological studies that examined and quantified neural precursor cells and immature neurons based on marker gene expression in post-mortem human hippocampal tissue^22–25^. However, despite accumulating evidence supporting the presence of a sizeable population of immature neurons throughout adult life^22,24,25^, the origin of these neurons and the extent of neurogenesis in the adult human hippocampus continues to be debated^26–29^.

As true for many ground-breaking biomedical discoveries, the field of adult neurogenesis has also oscillated from discovery to initial scepticism, and from convincing evidence in animals to deliberation around its occurrence and significance in the human brain. However, a feature of adult hippocampal neurogenesis that has intrigued several neuroscientists is the unique physiology of adult-born neurons. These neurons exhibit heightened intrinsic excitability and a lower activation threshold^30–32^, particularly during their immature phase, which is believed to confer a high degree of plasticity and modulate excitation/inhibition balance with the hippocampal circuitry. Therefore, it is not surprising that considerable efforts have been devoted to answering two major questions. (i) how is adult hippocampal neurogenesis regulated? and (ii) what is its functional role?—with a view to harnessing this form of neural plasticity for promoting emotional and cognitive functions in various neurological conditions.

The mammalian hippocampus is densely packed with stress hormone receptors and is the key brain region involved in both sensing and regulating the response to stress^33^. Chronic and uncontrollable stress is a major risk factor for a wide range of neuropsychiatric disorders, including anxiety and depression^34^. In this review, we focus on the significance of adult hippocampal neurogenesis in mood regulation, evaluating the role of adult-born hippocampal neurons in mediating stress response and recovery. Although strong links exist between exposure to chronic stress, impairment in adult hippocampal neurogenesis and anxiety- and depression-like behaviour^35^, the nature of the relationship between this form of cellular plasticity and mood-related disorders requires further clarification. Here, we appraise studies that have directly examined the causal role of adult-born hippocampal neurons in altering mood-related behaviour in rodent models. These include both loss-of-function studies that have used ablation approaches and gain-of-function studies that have adopted genetic strategies to probe the function of these cells under baseline conditions as well as following stress and antidepressant treatment. We consider the strengths and limitations of these approaches in resolving the debate around whether adult-born neurons in the hippocampus confer resilience or promote susceptibility to stress-induced mood dysfunction. Lastly, we propose a unifying framework that may help to unravel the mechanisms that link stress-induced changes in adult hippocampal neurogenesis to affective behaviour, thereby paving the way for the development of novel antidepressant and/or resilience-promoting therapeutics.

## Regulation of adult hippocampal neurogenesis by stress and antidepressants

Stress and clinical antidepressants have been identified as major regulators of adult hippocampal neurogenesis, with stress generally decreasing and antidepressants increasing the number of newborn neurons. Landmark studies in the 1990s by Gould and colleagues revealed that exposure to stressors decreases cell proliferation in the adult hippocampus^36,37^, an effect that was postulated to be driven by increases in the release of the stress hormone, glucocorticoid. In support of this notion, adrenalectomised animals showed an increase in cell proliferation^38,39^, whereas treatment with corticosterone (the major glucocorticoid in rodents) decreased cell proliferation^38^ in the hippocampus. Since these initial findings, numerous studies have reported a decline in adult hippocampal neurogenesis following exposure to various stressors during the prenatal or early postnatal periods, as well as in adult life (reviewed in ref. ^40^). A decrease in hippocampal neurogenesis has commonly been observed across multiple stress paradigms including chronic unpredictable mild stress^41,42^, social defeat stress^43^, and restraint stress^41,44^ as well as following chronic administration of corticosterone^45,46^. Besides affecting cell proliferation, chronic stress has been shown to impair neuronal differentiation and decrease the survival of newborn neurons^43,47^. However, there have also been studies that show no effects of stress on hippocampal cell proliferation^48–51^, with some even reporting an increase in hippocampal neurogenesis following stress^52–54^. Overall, stress-induced impairments in adult hippocampal neurogenesis appear to be influenced by multiple factors such as the nature and duration (acute versus chronic) of the stressors, and species, sex and strain of the animal.

The results demonstrating the deleterious effects of stress on adult hippocampal neurogenesis raised the question of whether antidepressant treatment could reverse or ameliorate these effects^55^. The first evidence appeared in 2000 from the Duman laboratory^56^, who demonstrated that the administration of different classes of antidepressants such as a monoamine oxidase inhibitor (tranylcypromine), a selective serotonin reuptake inhibitor (fluoxetine), and a selective norepinephrine reuptake inhibitor (reboxetine), as well as electroconvulsive therapy, led to an increase in cell proliferation and enhancement of adult hippocampal neurogenesis. These findings were replicated in several subsequent studies which highlighted the requirement of chronic but not acute administration of these clinical antidepressants to produce the neurogenic effect^56–59^. More recently, the fast-acting antidepressant ketamine has also been shown to enhance hippocampal neurogenesis by accelerating the maturation of adult-born neurons^60^. Similar to the effects of stress, these antidepressants have been shown to regulate different stages of adult neurogenesis, from stimulating the proliferation of neural precursor cells^61^, to promoting the survival and accelerating the maturation of the newborn neurons^60,62–64^. The neurogenesis-enhancing effects of antidepressants have also been found to be evolutionarily conserved, with post-mortem studies showing increases in cell proliferation in the hippocampi of depressed patients treated with antidepressants^65^. Interestingly, as opposed to the neurogenesis-promoting effects of antidepressants, chronic administration of diazepam, a commonly prescribed anxiolytic, has been shown to have no effects on the levels of adult hippocampal neurogenesis^66,67^.

## Evidence for a causal link between adult hippocampal neurogenesis and mood regulation

Evidence showing that chronic stress exerts an inhibitory effect on adult neurogenesis^36,38^ and is a major risk factor for the development of mood disorders^68^ together with data showing neurogenesis-enhancing actions of antidepressants^56^ led to the neurogenic theory of anxiety/depression^69^, which posits that stress-induced impairment in neurogenesis is an important causal factor in the aetiology of anxiety/depression and that restoration of neurogenesis is necessary for the therapeutic effects of antidepressants.

To test this theory, various approaches including chemical^70–72^, X-ray irradiation^45,73^, and genetic strategies^41,74,75^ were developed to ablate adult neurogenesis in rodents (summarised in Table 1). In naïve non-stressed animals, most studies using methylazoxymethanol acetate (MAM), a mitotic blocker, or focal X-irradiation, which efficiently ablates adult neurogenesis reported no changes in anxiety/depression-related behaviours (see Box 1). However, findings from studies using transgenic rodent models in which adult neurogenesis was abrogated by expressing ‘toxic’ genes under neural precursor cell promoters such as the *glial fibrillary acidic protein* (*GFAP*) or *Nestin* have proven more variable. For example, in mice expressing a viral form of the enzyme thymidine kinase (TK) under the control of *GFAP*, ganciclovir treatment led to a near-complete abrogation of adult hippocampal neurogenesis^41^. An increase in anxiety-like behaviour in the forced swim test but not in the elevated plus maze, open field test or light/dark test was noted in these neurogenesis-ablated mice. On the other hand, expression of the proapoptotic protein Bax in Nestin-Bax mice led to increased anxiety-like behaviour in the elevated plus maze and light/dark test but not in the novelty-suppressed feeding test^74^, whereas inducible expression of the Diptheria toxin A chain (DTA) in an inducible Nestin-Cre mice resulted in increased latency to eat in the novelty-suppressed feeding test but not in the light/dark test^75^. The emergence of anxiety-like behaviour in naïve, non-stressed mice was therefore evident in select behavioural tests and was specific to genetic models used for ablating adult neurogenesis. In contrast, a study using inducible deletion of *Tbr2*, a T-box transcription factor highly expressed in neural precursor cells to block adult neurogenesis reported a reduction in anxiety-like behaviour in neurogenesis-ablated compared to control mice, but only when these tests were conducted during the dark cycle^76^.

**Table 1:** Effects of ablating adult neurogenesis on mood-related behaviours in naïve, non-stressed animals.

Ablation approach	Species	Hippocampus-specific ablation?	Behavioural tests	Increased depressive/anxious behaviours?	Reference
MAM	Rats	No	Novelty-suppressed feeding	Yes	Bessa et al., 2009^70^
			Sucrose preference test	No	Bessa et al., 2009^70^
			Sucrose consumption	No	Jayatissa et al., 2010^71^
			Forced swim test	No	Bessa et al., 2009^70^
			Elevated plus maze	No	Shors et al., 2002^72^
X-irradiation	Mice	Yes	Novelty-suppressed feeding	No	David et al., 2009^45^; Santarelli et al., 2003^73^; Wang et al., 2008^64^
		Yes	Sucrose consumption	No	Noonan et al., 2010^106^
		Yes	Forced swim test	No	Holick et al., 2008^107^; David et al., 2009^45^
		Yes	Elevated plus maze	No	Saxe et al., 2006^108^
		Yes	Social interaction/avoidance	Yes	Lagace et al., 2010^52^
		Yes	Open field test	No	Saxe et al., 2006^108^; David et al., 2009^45^; Fuss et al., 2010^109^
		Yes	Light/Dark test	No	Saxe et al., 2006^108^
				Yes	Fuss et al., 2010^109^
	Rats	Yes	Novelty-suppressed feeding	No	Zhu et al., 2010^84^
		Yes	Sucrose consumption	No	Noonan et al., 2010^106^
			Forced swim test	No	Airan et al., 2007^110^; Zhu et al., 2010^84^
GFAP-TK	Mice	No	Forced swim test	Yes	Snyder et al., 2011^41^
			Sucrose preference test	Yes	Snyder et al., 2011^41^
			Elevated plus maze	No	Snyder et al., 2011^41^
		No	Open field test	No	Schloesser et al., 2010^43^
			Light/Dark test	No	Schloesser et al., 2010^43^
GFAP-TK	Rats	No	Novelty-suppressed feeding	No	Snyder et al., 2016^41^
			Sucrose preference test	Yes	Snyder et al., 2016^41^
			Open field test	No	Snyder et al., 2016^41^
Nestin-Bax	Mice	No	Novelty-suppressed Feeding	No	Revest et al., 2009^74^
			Forced swim test	No	Revest et al., 2009^74^
			Elevated plus maze	Yes	Revest et al., 2009^74^
			Light/Dark test	Yes	Revest et al., 2009^74^
Nestin-TK	Mice	No	Tail suspension test	No	Singer et al., 2009^111^
Nestin-Cre^ERT2^/floxed DTA	Mice	No	Novelty induced hypophagia	Yes	Yun et al., 2016^75^
			Open field test	No	Yun et al., 2016^75^
			Tail suspension test	Yes	Yun et al., 2016^75^
			Light/Dark test	No	Yun et al., 2016^75^

Taken together, the role of adult-born hippocampal neurons in the regulation of baseline anxiety- or depression-related behaviour remains equivocal. Future studies using more specific strategies that selectively target adult-born neurons in the hippocampus while sparing those in other neurogenic niches in the brain are therefore needed. A recent study employing optogenetic activation of adult-born hippocampal neurons reported no changes in baseline anxiety-like behaviour^77^.

Compared to studies which investigated whether the loss of adult-born neurons is sufficient to induce anxiety- and depression-like behaviour, the findings from those which examined whether adult hippocampal neurogenesis is required for antidepressant efficacy have been more consistent. Using head-focused X-rays, Hen and colleagues were the first to show that the ablation of adult hippocampal neurogenesis blocked some of the beneficial effects of antidepressants fluoxetine and imipramine, in particular, their anxiolytic effects which were evaluated using the novelty-suppressed feeding test^73^. These findings were replicated by several other studies which used different approaches to ablate adult neurogenesis^45,64,78,79^. Recently, Tunc-Ozcan et al.^80^ used a chemogenetic approach to selectively modulate the activity of adult-born neurons and found that silencing their activity using Gi-coupled DREADDs (designer receptors exclusively activated by designer drugs) blocked the efficacy of fluoxetine in the tail suspension and open field tests. However, it is also worthwhile noting that the actions of antidepressants have been shown to be mediated via both neurogenesis-dependent and -independent mechanisms^45,78,81–83^.

Box 1 Behavioural tests assessing anxiety- and depression-like behaviour in rodents Assessing externally measurable anxiety- and depression-related phenotypes in rodents remains a major challenge, with widely used behavioural tasks recapitulating only aspects of these conditions. Various strategies exhibiting face, predictive and construct validity have now been developed^112^. For rodents, environments such as bright-lit, open exposed spaces where they are more visible to predators, are innately recognised as harbouring threats and avoided. These environmental features (open field) have therefore been used in the laboratory setting to elicit avoidance behaviour as a proxy for anxiety state. Approach–avoidance tasks exploiting conflicting motivational states such as measuring the rodent’s exploratory drive against the innate drive to avoid danger^113^ have been developed to assess levels of anxiety. Such features are present in tasks such as the novelty-suppressed feeding, elevated plus maze, and light/dark box test. A major advantage of these tasks is that they require no prior training, thereby assessing animals’ innate valence without the confounding effects of extensive learning. Similarly, measuring symptoms associated with depression, which is a complex, multifactorial, and heterogenous clinical syndrome in rodents has been difficult. However, some of the symptoms commonly observed in human patients are measured as depression-like behaviour in rodents. These include social avoidance which is measured following a chronic social defeat stress paradigm which induces avoidance behaviour in susceptible animals, anhedonia which is measured using a sucrose preference test, and learned helplessness and passive coping which are measured using a forced swim test and tail suspension test.

## Do adult-born hippocampal neurons buffer or increase susceptibility to stress-induced mood disorders?

To fully realise the potential of adult hippocampal neurogenesis as a therapeutic strategy for stress-induced mood disorders, several studies have explored whether and how adult-born neurons in the hippocampus mediate the behavioural and endocrine response to chronic stressors (summarised in Table 2). Most studies using nonspecific ablation strategies such as cranial X-irradiation or cytostatic agents have reported no changes in the susceptibility to stress-induced anxiety- or depression-related behaviours^43,45,78,84–86^. One exception was the study by Bessa and colleagues which showed that treatment with the cytostatic agent MAM during the last two weeks of a 6-week chronic mild stress paradigm exacerbated stress-induced anxiety-like behaviour in the novelty-suppressed feeding test^70^, suggesting the stress-buffering actions of adult hippocampal neurogenesis. However, adult neurogenesis has also been implicated in promoting susceptibility to stress-induced mood dysfunction^52,87^. Using cranial X-irradiation ref. ^52^ found that ablation of adult neurogenesis prevented social defeat stress-induced social avoidance behaviour in mice. In another study, fluoxetine-induced enhancement of hippocampal neurogenesis was found to increase behavioural vulnerability, and blockade of neurogenesis using MAM was shown to protect against maladaptive behavioural changes induced by stress re-exposure^87^.

**Table 2:** Effects of ablating adult neurogenesis on mood-related behaviours in stressed animals.

Ablation methods	Start of ablation	Stress model	Behavioural Test	Effects of ablation on stress-induced changes in behaviour	Reference
X-irradiation	The 22nd day of stress	5 weeks of UCMS	Coat score	No effects	Zhu et al., 2010^84^
			Novelty-suppressed feeding	No effects	
	5 weeks before stress	5 weeks of UCMS	Splash test	No effects	Surget et al., 2008^78^
			Novelty-suppressed feeding	No effects	
	5 weeks before stress	10 days of social defeat stress	Social interaction test	Blocked stress-induced social avoidance	Lagace et al., 2010^52^
	1 week before stress	4 weeks chronic low CORT	Open field test	No effects	David et al., 2009^45^
			Novelty-suppressed feeding	No effects	
			Forced swim test	No effects	
MAM	The 4th week of chronic stress	6 weeks of UCMS	Forced swim test	No effects	Bessa et al., 2009^70^
			Sucrose preference test	No effects	
			Novelty-suppressed feeding	Aggravated stress-induced anxiety-like behaviour	
GFAP-TK animal models	Before stress	14 days of social defeat stress	Sucrose preference test	No effects	Schloesser et al., 2010^43^
			Light-dark box	No effects	
			Social interaction test	No effects	
	The start of stress	4 weeks of unpredictable restraint stress	Novelty-suppressed feeding	A trend of decreased anxiety-like behaviour in stressed mice	Schoenfeld et al., 2017^89^
			Sucrose Preference Test	No effects	

On the other hand, studies using genetic strategies have reported that adult hippocampal neurogenesis buffers the stress response such that blocking neurogenesis leads to an enhanced glucocorticoid response and an increase in anxiety- and depressive-like behaviour^41,82^. Corroborating these results, a recent study found that chemogenetic silencing of adult-born neurons specifically in the ventral dentate gyrus increased the susceptibility to social defeat stress, resulting in avoidance of a novel mouse in a social interaction test^88^. However, as opposed to these findings supporting the stress-buffering actions of adult-born hippocampal neurons, a decrease in stress-induced anxiety-like behaviour has also been reported following blockade of adult neurogenesis in GFAP-TK rats^89^.

Based on these conflicting findings, the question of whether adult-born neurons in the hippocampus buffer or exacerbate the behavioural and endocrine effects of stress remains unresolved. Several factors may contribute to these discrepant results, including the ablation strategy and its efficacy and selectivity, the type of stressor and the timing and duration of both the stressor and ablation. For example, X-irradiation which robustly ablates hippocampal neurogenesis has also been shown to induce inflammation and morphological and synaptic alterations that take a long time to fully recover^52,70,78,90^. Moreover, it has been also shown to impact oligodendrocyte progenitor cells in the brain—a cell population that regulates anxiety and depressive-like behaviours in rodents^91,92^. While transgenic rodent models (GFAP-TK or Nestin-TK) have provided a more controlled approach to ablate adult neurogenesis, they also require prolonged ganciclovir treatment lasting up to several weeks to achieve good ablation efficiency^43^. In addition to ablating neural precursor cells, the addition of new glial cells is also impaired in these models^93^ which may further confound interpretations given the important roles of these cells in the pathophysiology of major depression^94–96^. Another important confound is that global manipulations of neurogenesis affect other neurogenic areas such as the subventricular zone which in turn likely impair olfactory functions which are known to influence emotional states^97,98^. These limitations highlight the need to use specific approaches with spatial and temporal control to either deplete or regulate the activity of adult-born neurons in the hippocampus in order to define their role in mediating the stress response and/or recovery.

## Is enhancement of adult hippocampal neurogenesis sufficient to improve mood-related behaviour?

Compared to loss-of-function studies, very few investigations have examined whether selectively increasing adult hippocampal neurogenesis is sufficient to improve mood-related behaviour. A gain-of-function approach involving neural precursor cell-selective deletion of the proto-oncogene Bax was developed to genetically expand the population of adult-born neurons^99^. Although no baseline improvement in mood was found, enhancing the level of neurogenesis using this approach was shown to promote behavioural resilience to chronic corticosterone administration^46^ and chronic social defeat stress^88^. However, these results need to be considered carefully given that Bax has a non-apoptotic role in the regulation of synaptic plasticity^100^. Interestingly, a recent study showed that chemogenetic stimulation of adult-born neurons alleviates depressive-like behaviour following uncontrolled chronic mild stress in mice^80^, indicating that the activity of these neurons is sufficient to promote stress resilience.

## Concluding remarks and outlook

The connection between stress, adult hippocampal neurogenesis and neuropsychiatric disorders has been a subject of intense investigation over the past two decades. However, so far, the neurogenic theory of mood disorders has fallen short in providing a unified mechanism that links stress to affective behaviour, as simple subtraction or addition of adult-born neurons alone is not sufficient to lead to anxiety/depression or have antidepressant-like effects, respectively. Moreover, the jury is still out regarding whether and how adult-born neurons in the hippocampus contribute towards promoting resilience or susceptibility to stress-related mood disorders.

Several important considerations have emerged from these studies. First, a change in the absolute number of adult-born hippocampal neurons alone is unlikely to be the key factor linking stress to altered behaviour. Instead, the activity of these neurons could be a major determinant in unravelling their role in the regulation of stress-induced changes in mood-related behaviour. Second, off-target effects of ablation approach used in the majority of studies, including the chronic nature of manipulations which likely lead to compensatory mechanisms as well as brain-wide ablation, as in the case of genetic strategies, preclude determining the precise role of adult-born hippocampal neurons. A third important consideration is the nature and duration of the stressors modelled in these studies, which are likely to influence the hypothalamic-pituitary-adrenal axis differently, resulting in stressor-specific alterations in the neurotransmitter and neuropeptide signalling in the brain. These may differentially impact various stages of adult hippocampal neurogenesis. Fourth, the timing of ablation is also a critical variable, with most studies examining the effects of stress on mood-related behaviour in neurogenesis-null animals. Characterising stress-induced changes in the properties of adult-born neurons and evaluating their causal role during different phases (acute, sub-chronic, chronic) of stress is likely to provide significant insights into the role of these neurons in mediating stress susceptibility versus resilience. Finally, not all mood-related behaviours are equally impacted following manipulation of adult hippocampal neurogenesis in rodents, with greater effects seen on anxiety-like (such as approach–avoidance) behaviour compared to depression-related (such as passive coping, anhedonia). Even amongst approach–avoidance type behaviour which is evaluated using the elevated plus maze, novelty-suppressed feeding or social avoidance tasks, differential effects of adult neurogenesis manipulations have been reported. It is likely that these may be driven by differences in exploration, motivation and reward contingencies associated with these tasks, which have distinct underlying neural circuits. Therefore, unravelling specific neural circuits that are modulated by adult hippocampal neurogenesis is an important direction for future studies. As stress-associated neuropsychiatric disorders are complex and heterogeneous in symptom criteria, defining the role of adult-born hippocampal neurons in the regulation of specific behaviour may therefore aid in stratifying patients who are likely to benefit from neurogenesis-based therapeutic strategies. Moreover, changes in cognitive processes such as spatial and contextual learning and memory that are modulated by adult-born neurons are not usually examined in these models but are likely to contribute towards differences in susceptibility versus resilience to stress-related mood disorders^15,19^.

Another key consideration which has largely been overlooked in previous studies is the relative contribution of adult-born neurons at immature versus mature stages of their development in both encoding and mediating the response to stressors^101^. The connectivity of adult-born neurons within the dentate gyrus—CA3 circuit at different stages of their morphological and functional maturation is complex but is beginning to be unravelled^102,103^. In particular, adult-born neurons exhibit a critical period of heightened intrinsic excitability and synaptic plasticity during their immature phase (approximately 4–6-week-old neurons) with the capacity to profoundly influence the activity of mature neurons within the dentate gyrus, thereby influencing the local circuit to control behaviour^88^. Therefore, unravelling how stress modifies intrinsic excitability as well as the presynaptic and postsynaptic connectome of adult-born neurons would shed further light on their contribution to the hippocampal circuit regulation and the control of behaviour.

Based on collective findings from previous studies, our working hypothesis is that the unique properties of adult-born hippocampal neurons during their critical period of maturation are functionally important and causally link chronic stress to altered mood-related behaviour. We posit therefore that (i) chronic stress exposure alters the cellular and physiological properties of these neurons, and their pattern of connectivity, resulting in a dysfunctional hippocampal circuit and that (ii) antidepressants exert their beneficial effects, in part, by modulating distinct cellular and functional properties of immature adult-born hippocampal neurons.

Thus, future studies should move beyond altering the overall levels of adult hippocampal neurogenesis and instead focus on monitoring and manipulating stress-induced changes in the molecular, cellular, and physiological properties of adult-born neurons as well as mapping their connectome. Also, given the functional dichotomy between dorsal and ventral hippocampal functions^104^ with the ventral hippocampus preferentially involved in mood regulation, future research should consider conducting region-specific manipulation of adult hippocampal neurogenesis to better define their role in regulating specific aspects of mood-related behaviour. This coupled with approaches that allow spatial and temporal control to both visualise and modulate the activity of newborn neurons at various stages of their maturation during a behavioural task, would help reveal their contribution to the stress response, recovery, and mood regulation. Whilst historically male rodents have been used in most studies, given the higher incidence of stress-related mood disorders in women than men, future studies should investigate the role of adult-born hippocampal neurons in both sexes. Recently, there have been some efforts towards developing new chronic stress paradigms that are effective in both adult male and female rodents in inducing mood-related behavioural changes^105^.

Collectively, the findings from this integrated approach are likely to provide important new perspectives on the physiological and functional significance of adult-born hippocampal neurons in mediating the stress response. This would bring us closer to developing treatments for stress-related mood disorders that are founded on a deeper molecular and cellular understanding of a defined neural subtype and circuit.

### Reporting summary

Further information on research design is available in the Nature Research Reporting Summary linked to this article.

## Supplementary information

Reporting Summary

## Data Availability

Data sharing not applicable to this article as no datasets were generated or analysed during the current study.
